# 3D tumor spheroid models for *in vitro* therapeutic screening: a systematic approach to enhance the biological relevance of data obtained

**DOI:** 10.1038/srep19103

**Published:** 2016-01-11

**Authors:** Michele Zanoni, Filippo Piccinini, Chiara Arienti, Alice Zamagni, Spartaco Santi, Rolando Polico, Alessandro Bevilacqua, Anna Tesei

**Affiliations:** 1 Biosciences Laboratory, Istituto Scientifico Romagnolo per lo Studio e la Cura dei Tumori (IRST) IRCCS, Meldola, Italy; 2 Advanced Research Center on Electronic Systems for Information and Communication Technologies “E. De Castro” (ARCES), University of Bologna, Italy; 3 Department of Computer Science and Engineering (DISI), University of Bologna, Italy; 4 Institute of Molecular Genetics, CNR-National Research Council of Italy, Bologna, Italy; 5 SC Laboratory of Musculoskeletal Cell Biology, Rizzoli Orthopedic Institute (IOR) IRCCS, Bologna, Italy; 6 Radiotherapy Unit, IRST IRCCS, Meldola, Italy

## Abstract

The potential of a spheroid tumor model composed of cells in different proliferative and metabolic states for the development of new anticancer strategies has been amply demonstrated. However, there is little or no information in the literature on the problems of reproducibility of data originating from experiments using 3D models. Our analyses, carried out using a novel open source software capable of performing an automatic image analysis of 3D tumor colonies, showed that a number of morphology parameters affect the response of large spheroids to treatment. In particular, we found that both spheroid volume and shape may be a source of variability. We also compared some commercially available viability assays specifically designed for 3D models. In conclusion, our data indicate the need for a pre-selection of tumor spheroids of homogeneous volume and shape to reduce data variability to a minimum before use in a cytotoxicity test. In addition, we identified and validated a cytotoxicity test capable of providing meaningful data on the damage induced in large tumor spheroids of up to diameter in 650 μm by different kinds of treatments.

Chemotherapy, together with surgery and radiotherapy, is one of most common types of cancer treatment. Since its introduction, considerable efforts have been made by clinicians and researchers to optimize drug efficacy and minimize side-effects. In parallel, the pharmaceutical industry has increased investments into drug discovery programs to provide new molecules and biologic agents for clinical development and pharma market. However, the attrition rate for cancer drugs entering early clinical trials has reached disturbing heights^1^, suggesting that preclinical development has not been successful in identifying agents that can modify the outcome of human cancer^2^. Furthermore, there is also substantial inter-patient variability in response to new generation drugs, and research into personalized medicine focusing on the development of predictive biomarkers and preclinical cytotoxicity models has yet to provide satisfactory results.

In the past, the *in vitro* screening of synthetic and natural product libraries for novel anticancer agents mainly relied on cytotoxicity assays using established cancer cell lines grown as two-dimensional (2D) cultures that exhibited a rapid, uncontrolled growth phenotype. Such an approach indisputably has several strengths and in the past has contributed significantly to increasing our knowledge of tumor biology and to stimulating research into the field of anticancer drug discovery and development. However, cytotoxicity assays based on 2D cell cultures show important limitations that may partially account for the high rate of clinical trial failures for new molecules notwithstanding excellent antitumor properties observed in both *in vitro* and *in vivo* preclinical testing^3^,^4^. In particular, conventional 2D cell cultures are not capable of mimicking the complexity and heterogeneity of clinical tumors as *in vivo* tumors grow in a three-dimensional (3D) conformation with a specific organization and architecture that a 2D monolayer cell culture cannot reproduce. Consequently, numerous signals that govern different cellular processes are lost when cells are grown in 2D plastic substrata^5^.

Three-dimensional (3D) growth of immortalized established cell lines or primary cell cultures is regarded as a more stringent and representative model on which to perform *in vitro* drug screening^6^. As reported in detail by Kimlin *et al.*^7^, 3D cell cultures possess several *in vivo* features of tumors such as cell-cell interaction^8^, hypoxia^9^, drug penetration^10^, response and resistance^9^, and production/deposition of extracellular matrix^7^. All of these factors shift growth dependence away from the phenotype of unrestrained proliferation which is dominant in standard 2D cultures. Furthermore, the study of cancer cell dynamics in a 3D context allows us to recapitulate the architecture of living tissue and to better investigate the pathobiology of human cancers^11^. It is now common opinion that *in vitro* 3D cultures could fill the gap between conventional 2D *in vitro* testing and animal models^12^, and many researchers recommend the use of 3D cell cultures in drug screening programs as support for conventional 2D monolayer studies and before activating animal protocols^13^,^14^.

Several types of 3D culture models have been developed. These are generally subdivided into liquid-based and scaffold-based 3D-models^6^. Scaffold platforms for 3D culture are made of synthetic or naturally-derived polymers that provide a support for cell growth and mimic extracellular matrix conditions. Currently available scaffolds often show difficulties in obtaining a controlled matrix^15^ that can support the cellular physiologic growth and interaction profile found *in vivo*^14^. Tumor spheroids are one of the most common and versatile scaffold-free methods for 3D cell culture. Spheroids are either self-assembling or are forced to grow as cell clusters starting from single cell suspensions^12^. Compared to cells cultured on a flat surface, they more closely mimic the complex scenario of tissues and organs where each cell interacts with nearby cells through the formation of desmosomes and dermal junctions^16^. Depending on the researcher’s needs and on the method used, it is possible to obtain spheroids of any dimension. In particular, large spheroids (starting from about 500 μm in diameter) are characterized by an external proliferating zone, an internal quiescent zone caused by limited distribution of oxygen, nutrients and metabolites, and a necrotic core^17^ resembling the cellular heterogeneity of solid *in vivo* tumors^18^,^19^,^20^,^21^.

The potential of spheroid models for the development of new anticancer strategies has been demonstrated over time^12^,^22^. Chemo- and radio-cytotoxicity are the most important areas of use for large spheroids^23^ as the clinical response to chemical or physical treatments also depends on parameters such as oxygen tension, compactness, apoptosis inhibition^24^, damage repair^25^, and permeability^26^. However, in addition to the fact that not all available methods produce an abundance of large tumor spheroids, the use of this model, composed of cells in different proliferative and metabolic states, has raised serious concerns about the reproducibility of data produced. Moreover, the method used to assess treatment effectiveness may be a source of variability as the conventional methods developed for 2D cultures^27^ are not suitable for 3D models^28^. The lack of a reference method has stimulated the development of new assays specifically designed for 3D models that appear to be more promising than the Trypan blue exclusion test, still the most widely used cytotoxicity test in this research field^28^.

In the present work we used AnaSP, a software suite written in MATLAB (©, The MathWorks, Inc., Natick, MA, USA) and distributed as an open-source tool at http://sourceforge.net/p/anasp/ 29. AnaSP was previously developed by our team to automatically analyze several morphological parameters of spheroids imaged with entry-level equipment such as a standard brightfield microscope. Our first goal was to show that spheroids heterogeneous in volume and shape constitute a potential source of variability and may respond differently to treatments. We would thus propose an approach based on spheroid pre-selection to obtain reproducible results using large spheroids (Fig. 1). Finally, we validated a viability assay capable of providing rigorous data about cytotoxic effects on spheroids of up to 650 μm in diameter.

## Results

### Comparison between different methods for producing large tumor spheroids

Before starting the analysis it is worth introducing the notion of “equivalent diameter”, needed in the presence of a non-perfect sphericity, and defined as the diameter of a circle having the same area as the spheroid section being imaged^29^. To establish the best and most reliable method of obtaining spheroids endowed with a diameter over 500 μm, we grew the same tumor cell line (A549) as 3D colonies by different methods (Table 1). Depending on the protocol used, we obtained tumor spheroids that differed in terms of morphology, dimension and abundance. Among the methods tested, only 2 produced a high number of 3D spheroids with a diameter over 500 μm, *i.e.* the “pellet culture” method, modified by us as reported in the Methods section, and the Rotary Cell Culture System (RCCS).

The pellet culture method enabled us to modulate spheroid dimension by varying the number of cells in the starting unicellular suspension. In particular, for A549 cell line we obtained spheroids with a diameter of 800–900 μm starting from a cellular suspension of 200,000 cells. We also obtained compact cellular aggregates within 24 hours of the initial centrifugation using this method. However, the high number of vials centrifuge tubes needed to obtain sufficient spheroids (one spheroid/tube) to fill a 96- or 384-well plate (one spheroid/well) commonly used for high-throughput cytotoxicity screening tests made the method unmanageable. The RCCS method permitted us to obtain a higher number of large spheroids starting from a relatively small number of cells. With regard to A549 cell line, we seeded 40 × 10^6^ cells in a single 50-ml vessel, obtaining 200–250 spheroids ranging from 500–1100 μm in equivalent diameter after 15 days.

### Volume and shape: a pre-selection based on morphological parameters

In our experience, all the different protocols tested produced spheroid populations of variable dimensions. ReViSP, an open-source software specifically developed to analyze the 3D volume of the spheroids was used to visualize their 3D surface starting from a brightfield image (http://sourceforge.net/p/revisp/)^30^. In addition, AnaSP was used to monitor different morphological parameters including volume and sphericity index (SI^31^). We selected spheroids with a similar volume to guarantee the homogeneity of our 3D population and placed them in a 96-well plate (one spheroid/well).

We also evaluated whether the shape of spheroids might affect the reproducibility of the experiments performed. With the exception of the pellet culture system, we found that the different protocols tested produced highly irregular-shaped 3D spheroids. Figure 2a shows a representative population of spheroids obtained with the RCCS method. Immediately after their formation, the spheroids showed high volume and shape variability. In particular, the most common spheroid shapes were spherical, ellipsoidal, Figure 8-shaped and irregular (Fig. 2b). However, the variability in sphericity was partially lost during the first week of culture (we called this period *spheroidization time*) in low-attachment plates, and a high number of spheroids (∼ 70%) acquired a spherical shape (SI ≥ 0.90). We monitored their shape over time and found that they maintained their round morphology over a 25-day culture period, in contrast to that observed for non-spherical spheroids. For example, in the ellipsoidal spheroids, the subset that most resembled the spherical population, we often observed substantial morphological changes due to cell detachment or budding of one or more small secondary spheroids (Fig. 2c). Such changes were more frequently detected in the other morphological subcategories (Fig. S1).

### Different shapes may reflect a different viability of the cells composing the spheroids

The darkest region of a spheroid imaged in brightfield is mainly composed of quiescent/dead cells (Fig. 3a)^22^. To further verify this, we used light sheet fluorescence microscopy (LSFM), an advanced method of fluorescence microscopy specifically developed for 3D structure mapping of large samples^32^. A549 spheroids were exposed to an ethidium-calcein mixture (with peaks in red and green wavelengths, respectively) for 30 minutes. Using LSFM, an optical section passing through the centre of the spheroid was then collected and used to calculate the intensity profile of the two fluorescences. Although both signals had a positive external zone, the red fluorescence highlighted a positive inner core composed of only red, non viable cells that corresponded to the darkest region in the brightfield image (Fig. 3b).

Notably, we observed that variations in spheroid shape were also accompanied by changes in the dimension of the inner core and in the thickness of the surrounding shell consists of proliferative, actively dividing cells (Fig. 3c). Accordingly, we hypothesized that the 3D shape reflects a different general viability of the spheroids. To better investigate this correlation, we selected 30 spheroids of similar volumes (0.112 ± 0.013 mm^3^) but belonging to the spherical (*n *= 15; SI ≥ 0.90) or non-spherical subtypes (*n* = 15; SI < 0.90) to analyze how different shapes influence the metabolic state of spheroids. The data obtained from the luminescence metabolic assay performed after one week of culture showed a significantly reduced viability of spherical spheroids with respect to the irregular-shaped group (P = 0.045) (Fig. 3d). This was probably due to a reduced distance between each cell and the culture medium interface in the non-spherical subset, leading to a wider zone of active cell proliferation.

### 3D viability assays

We aimed to identify the viability assay with the best performance to use with large spheroids. To this purpose, we tested three commercial assays, the Trypan blue exclusion test, the Perfecta3D-Cell Viability assay and the CellTiter-Glo®3D Cell Viability assay to evaluate the cytotoxic damage induced by a chemical or physical treatment.

In the first experiment we used A549 spheroids pre-selected for volume and shape and exposed for 72 hours to different concentrations of albumin-fenretinide nanocapsules (4-hydroxy(phenyl)retinamide, 4-HPR-HSA)^33^. Under brightfield inverted microscope, an evident disruption of the architectural structure of the spheroid population was observed as the 4-HPR-HSA dose increased. All the assays tested showed a decrease in cell viability in spheroids treated with 33 μM and 100 μM of 4-HPR-HSA. However, the data obtained with the Trypan blue exclusion test indicated high cytotoxicity starting from the lowest drug concentrations and a high level of data variability (average Coefficient of Variation –CV- 42.70) (Fig. 4a I ). Conversely, the Perfecta3D-Cell Viability assay and CellTiter-Glo®3D Cell Viability assay showed a dose-related efficacy of the drug, confirming the damage visualized under brightfield inverted microscope at the different concentrations tested (Fig. 4a II, III). In addition, the Perfecta3D-Cell Viability assay and CellTiter-Glo®3D Cell Viability assay showed a similar degree of data variability (average CV 7.53 and 7.23, respectively).

In the second experiment, A549 spheroids pre-selected for homogeneous volume and shape were exposed to different irradiation schedules for 5 consecutive days. The effect was evaluated 4 and 25 days after the end of treatment, the latter time span needed to detect irradiation damage in both *in vitro* experiments and clinical practice. Microscopic evaluation revealed a clear alteration in spheroid morphology starting 25 days after the end of the different irradiation treatments. The Trypan blue exclusion test confirmed the cytotoxic effect of the irradiation regimens from the 25^th^ post-treatment day onwards, albeit with some degree of variability (average CV 69.10 and 46.99 at 4 and 25 days from the end of treatment, respectively) (Fig. 4b I). The Perfecta 3D-Cell viability assay showed good reproducibility of the data (average CV 11.06 and 23.82 at 4 and 25 days, respectively but also highlighted damage starting 4 days after the end of radiation exposure that was not detected by either of the other two viability assays or by morphological analysis (Fig. 4b II, III). Finally, CellTiter-Glo®3D Cell Viability assay data showed low variability (average CV 8.62 and 5.86 at 4 and 25 days, respectively) and were in agreement with the results from light microscope analysis.

### LSFM imaging analysis validates viability data obtained with CellTiter-Glo®3D Cell Viability assay

To further strengthen the data obtained with CellTiter-Glo®3D Cell Viability assay, we investigated whether the bioluminescence induced by the test correlated with the viability status of all the cells composing the 3D spheroids, including those present in the inner core. To this purpose we selected 36 spheroids of MRC5 cells obtained by the pellet culture system. We opted for this system because of the short culture time needed to form compact spheroids with no or a very small central necrotic area. We subdivided the spheroids obtained into 5 volumetric categories: 0.025, 0.050, 0.100, 0.150, and 0.300 mm^3^ corresponding to an equivalent diameter of approximately 350, 450, 600, 650, and 850 μm, respectively (Fig. 5a).

First, we analyzed the viability of the 5 spheroid subsets, observing a linear increase (continuous red line in Fig. 5a) in the bioluminescence moving from spheroids of 350 to 650 μm in diameter, and a slight but significant deviance from linearity (green line) in spheroids with the highest biomass (diameter of about 850 μm). In parallel, we verified the capacity of the CellTiter-Glo®3D reagent solution to penetrate deeply into the spheroids. We exposed spheroids to a solution of Hoechst 33342 1 μg/ml or to a mixture composed of 50% of Hoechst 33342 and 50% CellTiter-Glo®3D reagent solution. The spheroids were imaged by LSFM after 30 minutes, the incubation time required for the cytotoxicity test (Fig. 5b). Hoechst 33342 alone showed a low degree of penetration in spheroids of about 650 μm in diameter (left spheroids), as confirmed by the nuclei staining of only the most superficial cell layers. Conversely, the mixture of Hoechst 33342 and CellTiter-Glo®3D reagent solution completely penetrated spheroids of the same dimensional category (right spheroids). We also investigated the capability of the CellTiter-Glo®3D reagent solution to penetrate larger spheroids (about 850 μm in diameter) but LSFM imaging analysis confirmed that only spheroids with a diameter up to 650 μm can be completely penetrated (Fig. 5c).

## Discussion

Tumor spheroid cultures have several unique features, *i.e.* they possess chemical gradients (oxygen, nutrients or catabolites) at diameters starting from 200 μm and develop a central secondary necrotic area from a diameter of 500 μm onwards. Cells located in the spheroid periphery reflect the *in vivo* situation of actively cycling tumor cells adjacent to capillaries while innermost cells become quiescent and eventually die via apoptosis or necrosis. However, the reliability of data furnished by these models is dependent on their use within a system that is carefully monitored to keep bias to a minimum. There are a number of critical issues associated with the use of these models, including the choice of the 3D culture method, the production of homogenous-sized spheroids, and the identification of the best cytotoxicity test to assess treatment efficacy.

**Figure 1:**
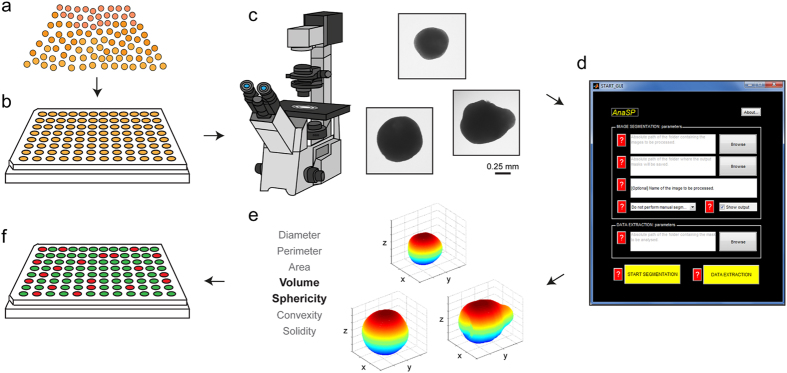
Schematic flow-chart of the image-based approach proposed to select a homogeneous population of large spheroids. (**a**) Spheroids of variable dimension and shape affect data reproducibility when they are used as an *in vitro* model to test drugs and radiotherapy treatments. (**b**) To select a sub-population of homogeneous spheroids, spheroids are seeded in low-attachment 96-well plates (one spheroid/well) and a brightfield image is acquired using an inverted widefield microscope (**c**). (**d**) AnaSP software (http://sourceforge.net/p/anasp/) can be used to automatically compute (**e**) several morphological parameters (3D reconstructions obtained by using ReViSP, http://sourceforge.net/p/revisp/). (**f**) A sub-population of homogeneous spheroids can be selected by analyzing volume and sphericity. The plate wells containing spheroids with similar volume and sphericity are shown in green.

**Figure 2:**
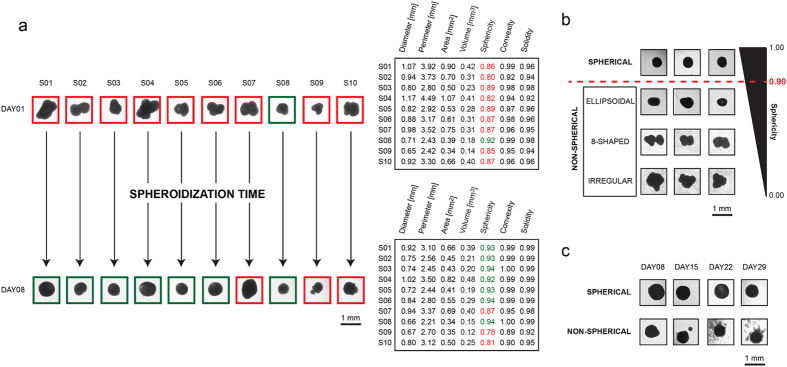
Spheroid-shape heterogeneity and evolution over time. (**a**) Very few spheroids generated by the RCCS method initially have a spherical shape (brightfield images of A549 3D cultures obtained using an Olympus inverted microscope with attached Nikon high speed DS-Vi1 colour digital camera, scale bar = 1 mm). After approximately one week of culture (*spheroidization time*), the majority can be considered as a real “spherical” spheroid (SI ≥ 0.90). (**b**) After the *spheroidization* period, a number of morphological classes of spheroids can still be observed: spherical, ellipsoidal, Figure 8-shaped and irregular. (**c**) We observed that the spherical-shaped spheroids generally maintain their morphology over time. Conversely, spheroids with a non-spherical shape after *spheroidization* are characterized by substantial morphological changes (*i.e.* cell detachment, budding of secondary spheroids).

**Figure 3:**
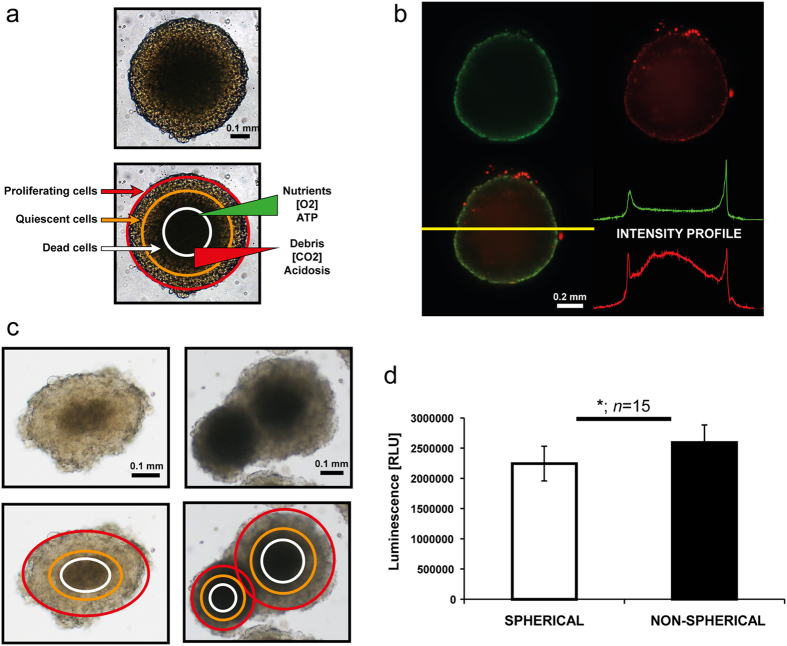
Relation between shape and viability of cells composing the spheroids. (**a**) Brightfield image of A549 spherical colony (top figure) and the same image with pathophysiological gradients schematically reported (bottom figure). Scale bar = 0.1mm. (**b**) Optical section of a live, spherical tumor spheroid obtained with light sheet fluorescence microscopy (LSFM, Lightsheet Z.1, Zeiss). Green = calcein-positive (live) cells; red = ethidium-positive (dead) cells. Scale bar = 0.2 mm. The fluorescence intensity profiles for both channels show the different distribution of live and dead cells in the spheroid structure; plots normalized on respective maximum value. (**c**) Variations in tumor shape are also accompanied by changes in the dimension of the inner core and in the thickness of the external layers mainly composed of actively proliferating cells. Brightfield images; scale bar = 0.1 mm. (**d**) Cell viability of tumor spheroids with homogeneous volume but different shapes (spherical *vs.* non-spherical) measured by CellTiter-Glo®3D Cell Viability assay. Bars = standard deviation (SD); means were calculated from a group of 15 spheroids/data set (*n*). *P = 0.045.

**Figure 4:**
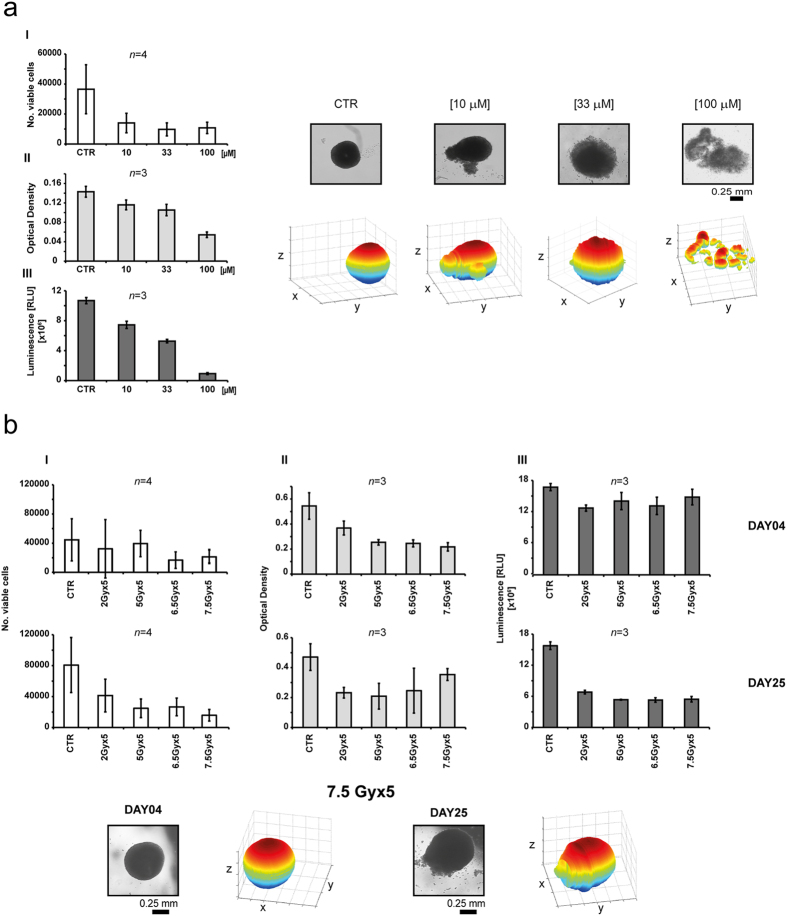
Evaluation of cell viability assay in tumor spheroid models. (**a**) Homogeneous-sized and -shaped A549 spheroids were treated for 72 h with three concentrations (10, 33 and 100 μM) of 4-HPR-HSA and viability was evaluated using 3 different assays: Trypan blue exclusion test, data are the mean of four repetitions, *n* = 4 (I), Perfecta3D-Cell Viability Assay, *n* = 3 (II), and CellTiter-Glo®3D Cell Viability assay, *n* = 3 (III). Brightfield imaging of untreated spheroids (control, CTR) and spheroids treated with increasing doses of 4-HPR-HSA (left to right) was acquired after a 72-h treatment; Scale bar= 0.25 mm. The corresponding 3D reconstructions were obtained by using ReViSP, http://sourceforge.net/p/revisp/. (**b**) Homogeneous-sized and -shaped A549 spheroids were exposed to four different radiation schedules (2 Gy × 5, 5 Gy × 5, 6.5 Gy × 5 and 7.5 Gy × 5 days). Cell viability was evaluated 4 and 25 days after the end of radiation treatment. Brightfield images of spheroids treated with 7.5Gy × 5 days were taken 4 and 25 days after the end of radiation treatment. Scale bar = 0.25 mm.

In the present work we used different protocols to create tumor spheroids (a 3D scaffold-free model), all showing various strengths and weaknesses. In our experience, the RCCS system was the most reliable at producing the highest number of large spheroids needed for the setting up of multiple multi-well plates for drug screening assays. The system easily formed 3D spheroids from several cell lines^34^,^35^ and, in our experience, from primary cultures of different tumor histotypes such as glioblastoma, ovarian carcinoma and melanoma. However, one weakness shared by all the systems studied was the production of a heterogeneous spheroid population in terms of volume and shape. Given the intense interest recently shown in the area of 3D tumor models, the problem of the morphological heterogeneity of spherical colonies has already been addressed. Several authors advise monitoring different morphological parameters such as diameter, perimeter, area, volume and sphericity, the variability of which may affect the reproducibility of the results obtained^31^,^36^,^37^,^38^,^39^. However, a lack of quantitative analytical methods makes these observations purely theoretical. To this purpose we developed software^30^, recently upgraded^29^, which is capable of accurately calculating several morphological parameters starting from a 2D image (Fig. 1). Using this tool, we found that both spheroid volume and spheroid shape generated by all the protocols may be a source of data variability. In particular, the spherical shape very rarely budded into secondary spheroids and was the most stable 3D structure. In addition, the selection of only spherically-shaped spheroids reduced variability caused by the different distribution of metabolic zones composing the 3D colonies. For example, in tumor spheroids with a highly irregular shape, it was not infrequent to observe the presence of 2 necrotic cores instead of only one. Such zones, as previously described, are constituted by cells with different proliferative status that may respond differently to chemical or physical treatments. In support of this, when we compared the viability of spheroids homogeneous in volume but varying in shape, we detected a statistically significant difference. This was probably due to the fact that the irregular morphology of the 3D colonies influenced the number of cells exposed to high levels of nutrients, oxygen and xenobiotics and, consequently, the percentage of actively proliferating cells.

**Figure 5:**
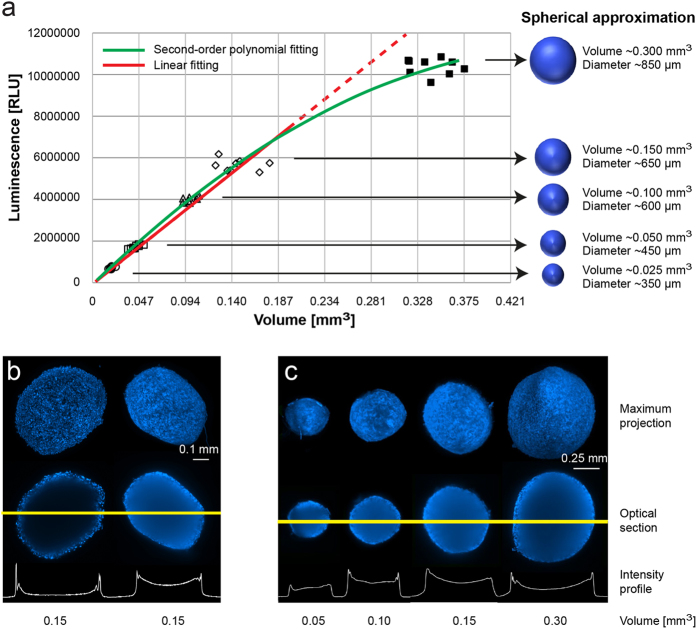
Validation of data obtained with CellTiter-Glo®3D Cell Viability assay in fibroblastic spheroids. (**a**) Spheroids were generated using the pellet culture system. The spheroids obtained were then subdivided into 5 volumetric categories: 0.025, 0.050, 0.100, 0.150, and 0.300 mm^3^, corresponding to an equivalent diameter of approximately 350, 450, 600, 650, and 850 μm. Using 9 homogeneous-shaped spheroids for each category, we performed the CellTiter-Glo®3D Cell Viability assay to investigate the relation between bioluminescence and volume. Bioluminescence increased in a linear manner up to a diameter of 650 μM (continuous red line). A significant deviance from linearity (green line) was observed for spheroids with a diameter of 850 μM. (**b**) Homogeneous-sized and -shaped MRC-5 spheroids were stained with Hoechst 33342 alone (left spheroids) or mixed with the CellTiter-Glo®3D reagent solution (right spheroids), as described in the Results section. Optical sections passing through the centre of the 3D structures and the corresponding maximum projections were captured with LSFM after 30 minutes. The fluorescence intensity profiles for the blue channel show a different degree of Hoechst 33342 penetration in the spheroid structures. Scale bar = 0.1 mm. (**c**) LSFM optical sections and fluorescence intensity profiles of spherical MRC-5 spheroids of increasing volume (left to right). The images show the complete penetration of Hoechst 33342 and CellTiter-Glo®3D reagent mixture in spheroids of up to 650 μm in diameter; Scale bar = 0.25 mm.

The choice of the method used to evaluate treatment-induced cytotoxicity is another critical issue. We tested several cytotoxicity assays and herein report the results obtained from the most promising methods. The best and most reproducible method to determine the viability of large spheroids for both chemical and for physical treatments was the CellTiter-Glo® 3D Cell Viability Assay based on luminescence reaction. To further confirm this result, we tested the assay on different-sized spheroids (diameter from 350 to 850 μm) with virtually no necrotic core to verify its capacity to provide information on the viability of the innermost cells. A linear increment in luminescence was observed as spheroid size increased, up to a maximum of 650 μm. A slight but significant non linearity observed in the final part of the regression curve (Fig. 5a) would seem to indicate a loss in the correlation between spheroid size over 650 μm and luminescence. Whilst this may be due to the incapacity of the reagent solution to penetrate spheroids of this size, it could, in our opinion, also be attributable to first the photon losses and spreading caused by refractive index mismatch within the depth samples that induced light distortions and scattering effects, second, non linear increase in cell viability caused by the presence of a necrotic core that reduced the number of viable cells.

In conclusion, the present work highlighted the importance of closely monitoring the morphological parameters of 3D tumor spheroids and of carefully selecting the most appropriate spherical colonies for use in cytotoxicity screening tests. To this purpose, we developed an affordable and user-friendly image-based approach to reduce bias to a minimum, a precondition for increasing data robustness prior to initiating expensive, time-consuming animal experiments. Further analysis of morphologic changes attributable to cytotoxic damage is needed to improve the accuracy of data, especially with regard to irradiation experiments.

## Methods

### Cell Culture

A549, a cell line derived from primary lung cancer, and MRC-5, a human fibroblast cell line derived from normal lung tissue, were purchased from the American Type Culture Collection (ATCC, Rockville, MD). A549 cell line was cultured in F12K (ATCC) supplemented with 10% FBS (Euroclone, Milan, Italy), 1% penicillin/streptomycin (GE Healthcare, Milan, Italy) and 2% amphotericin B (Euroclone). MRC-5 cells were maintained in EMEM (ATCC) supplemented with 10% FBS (Euroclone), 1% penicillin/streptomycin (GE Healthcare) and 2% amphotericin B (Euroclone). All the cell lines were checked periodically for mycoplasma contamination using the MycoAlertTM Mycoplasma Detection Kit (Lonza, Basel, Switzerland). Before seeding in the bioreactor culture vessels, cells were expanded and maintained as a monolayer at 37 °C and subcultured weekly. The same culture media used for the monolayer cultures were used to grow the cells as 3D colonies.

### Three-Dimensional Cell Culture Methods

#### Rotatory cell culture system

A rotatory cell culture system (RCCS) (Synthecon Inc., Houston, TX, USA) was used^35^. The rotator bases were placed inside a humidified 37 °C, 5% CO2 incubator and connected to power supplies set up externally to the incubator. All procedures were performed in sterile conditions under a laminar flow hood. Single cell suspensions of about 1 × 10^6^ cells/ml of A549 were placed in the 50-ml rotating chamber at an initial speed of 12 rpm. As the majority of cells formed aggregates and these aggregates gradually enlarged, speed was increased over time to avoid aggregate sedimentation within the culture vessels which could hinder complete spheroid formation. The culture medium was changed every 4 days and tumor spheroids with an equivalent diameter ranging from about 500–1300 μm (depending on the cell line used) were obtained in around 15 days. After the formation of the spheroids, the operator, working under the sterile laminar flow hood, transferred spheroids to 96-well low-attachment culture plates (Corning Inc., Corning, NY, USA) (one spheroid/well), each well previously filled with 100 μl of fresh culture medium.

#### Pellet culture system

This system is our adaptation of the pellet culture system previously described by Johnstone *et al.*^40^. Briefly, cells were detached from the flasks by trypsin, washed twice with PBS, counted and then resuspended in 500 μl of medium in 1.5-ml Eppendorf tubes at a concentration of 12.5 × 10^3^, 25 × 10^3^, 50 × 10^3^,100 × 10^3^ and 200 × 10^3^ cells/tube. The cellular suspensions were centrifuged at 500xg for 5 minutes. The cells, pelleted in tubes with loosened caps to permit gas exchange, were incubated at 37 °C, 5% CO_2_ for 24 hours. The spherical aggregates obtained (one spheroid/tube) were gently detached from the walls of the tubes and transferred to 96-well low-attachment culture plates (Corning) (one spheroid/well), ready for use.

#### Hanging drop culture method

A549 cells were cultured in hanging drops using Perfecta3D™ Hanging Drop Plates (3DBiomatrix, Inc., Ann Arbor, MI, USA) or the GravityPLUS^TM^ kit (InSphero AG, Schlieren, Switzerland), according to the manufacturers’ instructions. Cells were seeded at various cell densities to verify the possibility of obtaining spheroids of at least 500–600 μm in diameter (2 × 10^3^, 4 × 10^3^, and 6 × 10^3^ cells/well). In both assays, spheroidal colonies grew on the bottom of the wells after about 7 days’ culture at 37 °C in atmosphere containing 5%CO_2_.

#### Magnetic levitation method

The magnetic levitation method was used to obtain spheroids, as previously described by Haisler *et al.*^41^. Briefly, cells were cultured in advance to confluence (at least 70–80%) in 2D and, on the day before the start of the experiment, they were incubated with an 8 μl/cm^2^ magnetic nanoparticle assembly (Nano3D Biosciences Inc., Houston, Texas, USA) overnight to allow for cell attachment to the magnetic nanoparticles. The following day, the cells were detached and resuspended in medium in 24-well low-attachment culture plates (Corning Inc., Corning, NY,USA). In our study, different aliquots (1.5 × 10^3^, 3 × 10^3^, 6 × 10^3^, 12 × 10^3^ and 24 × 10^3^cells/well) of cells were seeded to define the optimal cell density needed to obtain spheroids with a diameter of about 500–600 μm. A magnetic drive was then placed atop the well plate to levitate the cells to the air-liquid interface, where the cells aggregated and interacted to form large 3D structures. After 4–5 days’ culture at 37 °C in an atmosphere containing 5% CO_2_, the spheroids were formed and ready to be used.

## Microscopy and Image Analysis

### Immunofluorescence and light sheet fluorescence microscopy (LSFM)

#### Sample preparation

For live imaging, spheroids of MRC-5 cell line were stained for DNA with Hoechst 33342 (Molecular Probes™). In particular, we exposed the spheroids to a solution of PBS 1x supplemented with Hoechst 33342 1 μg/ml. Viability of the A549 spheroids was measured with the LIVE/DEAD® Cell Viability Assay (Molecular Probes™). Briefly, samples were washed with warm phosphate-buffered saline (PBS), added to an ethidium-calcein mixture, and incubated for 30 min in a 37 °C incubator. After washing again with PBS, the spheroids were ready for the imaging procedure. All spheroids were mixed with low-melt agarose solution (Carl Roth GmbH) and the mixture was sucked into glass capillaries, with inner diameter of 1 mm. The agarose was allowed to gel at room temperature for five minutes before imaging.

#### Image analysis

LSFM permits the creation of a large, thin, uniform excitation light sheet with high 3D spatial resolution, good optical sectioning capability and minimal photobleaching and phototoxic effect. In practice, spheroid samples were imaged using Lightsheet Z.1 microscope (Carl Zeiss Microscopy GmbH, Jena, Germany) with 20x/1.0 detection optics and two-sided 10x/0.2 illumination optics equipped with two PCO EDGE 4.2 cameras (sCMOS sensor, square pixels of 6.5 μm side length, 2048 × 2048 pixel resolution, 16-bit grey level) (PCO AG, Kelheim, Germany). To counteract the degradation of the light sheet with high scattering spheroids, the specimen was sequentially illuminated through each of the two opposite illumination objectives, generating pairs of single-side illumination images which were instantaneously combined into optical sections with considerably improved penetration depth. The image sets were processed using ZEN imaging software (Carl Zeiss).

#### Morphological analysis of 3D tumor cultures

Growth and morphology of the 3D tumor colonies were monitored for several days as regards changes in area, volume and shape. Phase-contrast imaging and morphological analyses of spheroids were carried out with an inverted Olympus IX51 microscope (Olympus Corporation, Tokyo, Japan), equipped with a Nikon Digital Sight DS-Vi1 camera (CCD vision sensor, square pixels of 4.4 μM side length, 1600 × 1200 pixel resolution, 8-bit grey level) (Nikon Instruments, Spa. Florence, Italy). The open-source AnaSP^29^ and ReViSP^30^ software tools were used to achieve morphological 2D (diameter, perimeter, area) and 3D (volume, sphericity) parameters, and to select morphologically homogeneous spheroids, accordingly.

In particular, for each spheroid the volume was calculated by using ReViSP on a single phase-contrast image. The detailed description of the volume-estimation method implemented in ReViSP was previously described^30^. The main steps of the method can be briefly summarized as follow:

1. The analysed image is segmented to obtain a binary mask of the spheroid (*e.g.* by using AnaSP).
2. The binary mask is automatically checked and subdivided into the different parts composing the spheroid (*i.e.* main body and protuberances).
3. The 3D surface of each part is rendered by supposing a local symmetry around the maximum axis (technically called feret).
4. The external surface of the spheroid is obtained by connecting the single 3D parts through cylindrical connections locally adapted to follow the curvature of the objects to be connected.
5. The final volume is automatically estimated by counting the voxels bound by the surface and using the *x*-*y* pixel resolution coefficient to convert voxels to μM^3^.

The accuracy of the ReViSP was previously assessed by using several 3D synthetic models mimicking the real morphology of multicellular spheroids. However, by using the LSFM, we have now verified how ReViSP works also by directly using real multicellular spheroids (Supplementary Note).

The SI was calculated with AnaSP according to Equation 1^31^


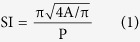


where A and P are area and perimeter of the spheroid.

## Chemical and Physical Treatments

### Drug

The albumin-fenretinide nanocapsules (4-hydroxy(phenyl)retinamide, 4-HPR-HSA) were prepared as previously described by Pignatta *et al.*^33^ and were freshly diluted in standard medium. 3D cell cultures of A549 were exposed to three drug concentrations (10 μM, 33 μM and 100 μM) for 72h. The albumin-fenretinide nanocapsules (4-hydroxy(phenyl)retinamide,4-HPR-HSA) was kindly provided by Prof. Isabella Orienti (FaBiT-Department of Pharmacy and Biotechnology, University of Bologna, Bologna, BO, Italy).

### Irradiation treatment

3D cell cultures of A549 were treated with 4 different radiation schedules (2 Gy × 5, 5 Gy  × 5, 6.5 Gy × 5 and 7.5 Gy × 5) using the linear acceleration Elekta Synergy Platform system (Elekta Oncology Systems, Stockholm, Sweden) and the irradiation system described by Tesei *et al.*^16^. Cell viability was evaluated 4 and 25 days after the end of the radiation treatment.

### Cell Viability Assays

#### Trypan blue exclusion test

3D aggregates were removed from the RCCS vessels and placed in single wells of a 96-well low-attachment culture plate. Each spheroid was harvested, disrupted using trypsin/EDTA 1 × (Euroclone, Milan, Italy), and Trypan blue solution 0.4% (Sigma, Milan, Italy) was used to stain the dead cells. Viable cells were then counted manually with a hemocytometer^42^.

#### Perfecta3D® Cell Viability Assay

(3DBiomatrix, Inc., Ann Arbor, MI, USA). The spheroids were removed from the 96-well low-attachment culture plate and placed separately in single wells of a 96-well culture plate (Corning Inc., Corning, NY,USA). WST-1 solution was added to each well. The optical density (OD) of treated and untreated cells was determined at a wavelength of 450 nm with a microplate reader after 4 hours’ incubation.

#### CellTiter-Glo® 3D Cell Viability Assay

(Promega, Milan, Italy). Homogeneous spheroids were removed from the 96-well low-attachment culture plate and placed separately in single wells of a 96-well opaque culture plate (BD Falcon). CellTiter-Glo® 3D reagent was added to each well and the luminescence signal was read after 30 minutes with the GloMax® bioluminescent reader (Promega).

### Statistical Analysis

After verification of normality of data by using Lilliefors and Jarque-Bera tests, difference among values observed were analyzed using the two-tailed Student’s t-test for unpaired observations. A P value < 0.05 was considered for statistical significance.

## Additional Information

**How to cite this article**: Zanoni, M. *et al.* 3D tumor spheroid models for *in vitro* therapeutic screening: a systematic approach to enhance the biological relevance of data obtained. *Sci. Rep.* **6**, 19103; doi: 10.1038/srep19103 (2016).

## Supplementary Material

Supplementary Information

## Figures and Tables

**Table 1:** Scaffold-free techniques suitable for obtaining tumor spheroid models.

	Landmark articles	§Time required [day]	§No. Cell required [x10^6^]	Equivalent diameter [μm] (range, mean±SD, CV, *n*)	Amount of spherical spheroids (SI ≥ 0.90)	Amount of large spheroids (diameter > 500 μm)
Magnetic Levitation	[ref. 41]	7	0.5	200–500, 347±87, 25.1, 28	Low	Low
Hanging Drop	[ref. 31]	7	0.6	200–500, 359±95, 26.5, 38	Low	Low
Pellet Cultures	[ref. 40]	1	20	800–900, 880±21, 2.4, 20	High	High
Rotating Wall Vessel (NASA Bioreactor)	[ref. 35]	15	40	500–1100, 897±98, 11.0, 192	Low*	High

Different methods were investigated for their ability to grow 3D spheroids starting from a single-cell suspension of the human NSCLC cell line A549. It was considered “high” an amount of spherical, or large spheroids, ≥50% of the total spheroids obtained by each specific method. Conversely, an amount <50% was considered “low”. SD = Standard deviation; CV = Coefficient of variation; *n* = number of spheroids analyzed; SI = Sphericity index;

^§^Time and number of cells needed to obtain sufficient spheroids to fill a 96-well plate;

^*^Before “*spheroidization time*”.
