# Supplementary material for: 3D tumor spheroid models for in vitro therapeutic screening: a systematic approach to enhance the biological relevance of data obtained
Source: Sci Rep. 2016 Jan 11;6:19103. doi: 10.1038/srep19103 (PMC4707510; doi:10.1038/srep19103)
Supplement: Supplementary Information [file srep19103-s1.pdf]

SUPPLEMENTARY INFORMATION

3D tumor spheroid models for in vitro therapeutic screening: a systematic approach to enhance the biological relevance of data obtained.

Michele Zanoni, Filippo Piccinini, Chiara Arienti, Alice Zamagni, Spartaco Santi, Rolando Polico, Alessandro Bevilacqua & Anna Tesei.

SUPPLEMENTARY FIGURE

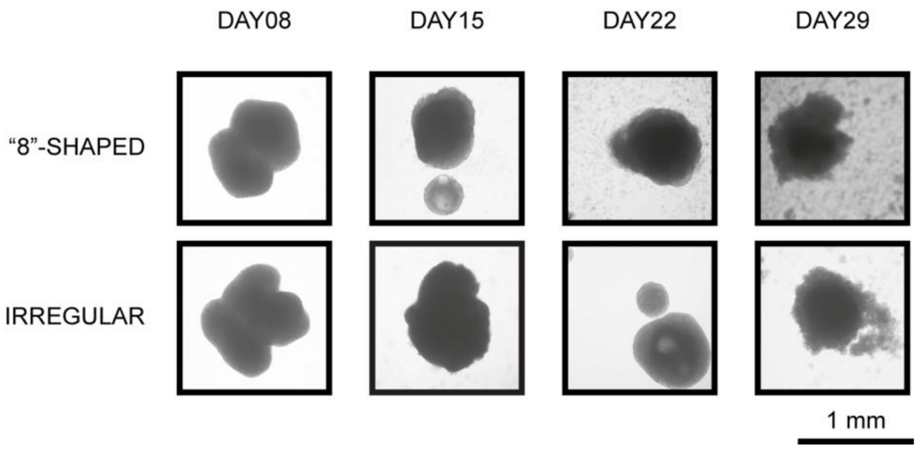

Figure S1.Figure 8-shaped and irregular spheroids after "*spheroidization time*" are characterized by substantial morphological changes (*i.e.* cell detachment, loss of structural integrity, budding of secondary spheroids).

SUPPLEMENTARY NOTE

VALIDATION OF *ReViSP* BY USING LIGHT SHEET FLUORESCENCE MICROSCOPY

Introduction

Reconstruction and Visualization from a Single Projection software (ReViSP, http://sourceforge.net/p/revisp/) is a single-image based method used to automatically estimate the volume of multicellular spheroids by simply processing a single 2D projection image. Estimating the volume from a single image is a challenging task, of course intrinsically prone to errors, due to a 2D image being just a projection of real 3D world. These errors substantially arise from the presence of asymmetries and protuberances hidden in the single projection analysed. However, if some priors are satisfied, the volume can be estimated with a good accuracy in real world applications. ReViSP requires the visualized object to be imaged when laying in a stable equilibrium position, and the spheroid to own a local symmetry around each symmetry axis defined for the different parts composing the spheroid (*i.e.* main body and protuberances). This condition is well-matched when a multicellular spheroid is seeded in a multi-well plate and it is imaged by using an upright or inverted optical widefield microscope. It is worth noting that ReViSP does not require fluorescent stains: the used projection image can be easily acquired keeping the spheroid living and using label-free microscopy techniques (*i.e.* using transmitted light or its modulations, such is in brightfield or phase-contrast). In practice, ReViSP allows to perform a contactless estimation of the volume of living multicellular spheroids.

While ReViSP had been already validated using 3D synthetic model spheroids built at a large scale, the light sheet fluorescence microscope (LSFM) gave us the chance to validate ReViSP with real spheroids. The LSFM allows scanning a spheroid along the z-axis, by illuminating several transaxial sections and acquiring the corresponding images. After aligning in z the different projections acquired, reconstructing the external surface of the visualized spheroid is simple, and the volume can be easily assessed by considering the x-y pixel resolution and the Δz step of the acquisitions between adjacent sections.

In order to compare the volume estimated by ReViSP with the *ground-truth* volume of the spheroids estimated by LSFM, three real multicellular spheroids were selected and the images of the sequence of sections were acquired by using the LSFM. In particular, to estimate the ground-truth volume of each spheroid we implemented the algorithm described in the next paragraph. Finally, we calculated the absolute error between the ground-truth volume and the volume of same spheroids estimated by using ReViSP.

Direct volume computation using LSFM

The current version of the software distributed with the LSFM (*i.e.* ZEN 2014 SP1 Black Edition) does not provide volume measurements. However, as an early assessment, the spheroid's volume was measured through an algorithm implemented in software to performing the following steps:

1) acquiring a sequence of sections achieved by scanning the spheroid along the *z*-axis
2) segmenting the images obtained to achieve black (background) and white (foreground) binary masks
3) computing each slice's volume as the product of the section area (number of white pixels, converted in micrometers using pixel resolution information) and the Δz acquisition step (distance between sections)
4) summing up all the slice volumes computed in 3), to achieve the final volume in μm^3

Materials and methods

In order to define the ground-truth volume of real multicellular spheroids, by imaging them with a LSFM, we built several A549 spheroids by using a Perfecta3D™ Hanging Drop Plate (3DBiomatrix, Inc., Ann Arbor, MI, USA), and we selected three different spheroids, with a maximum diameter size of 200 µm. Spheroids of this small size can be easily scanned by using a LSFM and the border of the spheroid is typically well defined in each image acquired. Hoechst 33342 was used to stain the cell's nucleus, Lamina immunostaining (secondary antibodies conjugated to FITC) for the cytoplasm. For the three spheroids 157, 150, and 105 sections were acquired, for each fluorescent dyes, with a Δ*z* of 0.760, 0.760, and 1.843 µm, respectively, and the signals of the fluorescence dyes were then fused in a single image. The ground-truth volume was then acquired using the procedure described above, where the binary masks were obtained by manually segmenting each fused image with *AnaSP* (http://sourceforge.net/p/anasp/).

In order to estimate the volume of the same spheroids by using ReViSP, for each sequence of the acquired fluorescence images, the maximum intensity projection was achieved. Subsequently, the binary mask was obtained by segmenting the maximum intensity projection image with AnaSP. Finally, the mask obtained was processed with ReViSP, and the *x-y* pixel resolution was used to convert voxels (considered with the three sides of equal length) to μm^3.

Results

In order to compare the spheroid's volume estimated by using a single projection, with the ground-truth volume (V_{GT}) obtained scanning in *z* the spheroid by using the LSFM, for each spheroid we computed the absolute error (*E*, expressed in percentage) according to Equation A:

$$E = \frac{\left|V_{\mathrm{GT}} - V_{\mathrm{ReViSP}}\right|}{mean\left(V_{\mathrm{GT}}, V_{\mathrm{ReViSP}}\right)} \cdot 100 \qquad \text{(A)}$$

V_{GT} and V_{ReViSP} are the volume of the same spheroid directly computed with LSFM and ReViSP, respectively. $|V_{GT} - V_{ReViSP}|$ is the absolute difference between V_{GT} and V_{ReViSP}. $mean(V_{GT}, V_{ReViSP})$ is the arithmetic mean between V_{GT} and V_{ReViSP}.

The *E* values obtained for the three spheroids used in the experiment decrease as their size increase, and are 8.8%, 8.3% and 3.4%, respectively, with an average *E*=6.8%.

Conclusions

The experiment performed gives us an estimate of the accuracy of ReViSP when used to estimate the volume of a multicellular spheroid by processing a single projection image. The errors obtained, ranging from 3.4% for the biggest spheroid to 8.8% for the smallest one, confirms that ReViSP represents an effective tool to compute the volume of living spheroids, simply visualized in brightfield.
